# Sequence characterisation and novel insights into bovine mastitis-associated *Streptococcus uberis* in dairy herds

**DOI:** 10.1038/s41598-021-82357-3

**Published:** 2021-02-04

**Authors:** Ben Vezina, Hulayyil Al-harbi, Hena R. Ramay, Martin Soust, Robert J. Moore, Timothy W. J. Olchowy, John I. Alawneh

**Affiliations:** 1 Good Clinical Practice Research Group (GCPRG), The University of Queensland, School of Veterinary Science, Gatton, QLD 4343 Australia; 2 The University of Queensland, School of Veterinary Science, Gatton, QLD 4343 Australia; 3 Centre for Cell Factories and Biopolymers, Griffith Institute for Drug Discovery, Griffith University, Nathan, Australia; 4 International Microbiome Centre, Cumming School of Medicine, University of Calgary, Calgary, AB Canada; 5 Terragen Biotech Pty Ltd., Coolum Beach, QLD 4573 Australia; 6 School of Science, RMIT University, Bundoora, Melbourne, 3083 Australia; 7 Faculty of Veterinary Medicine, University of Calgary, Calgary, AB T3R 1J3 Canada

**Keywords:** Bioinformatics, Genomic analysis, Clinical microbiology, Pathogens, Epidemiology, Genetics research

## Abstract

*Streptococcus uberis* is one of the most frequent mastitis-causing pathogens isolated from dairy cows. Further understanding of *S. uberis* genetics may help elucidate the disease pathogenesis. We compared the genomes of *S. uberis* isolates cultured from dairy cows located in distinctly different geographic regions of Australia. All isolates had novel multi locus sequence types (MLST) indicating a highly diverse population of *S. uberis*. Global clonal complexes (GCC) were more conserved. GCC ST86 and GCC ST143 represented 30% of the total isolates (*n* = 27) and were clustered within different geographic regions. Core genome phylogeny revealed low phylogenetic clustering by region, isolation source, and MLST. Identification of putative sortase (*srtA*) substrates and generation of a custom putative virulence factor database revealed genes which may explain the affinity of *S. uberis* for mammary tissue, evasion of antimicrobial efforts and disease pathogenesis. Of 27 isolates, four contained antibiotic resistance genes including an antimicrobial resistance cluster containing *mel*/*mef*(*A*), *mrsE*, *vatD*, *lnuD*, and transposon-mediated *lnuC* was also identified. These are novel genes for *S. uberis*, which suggests interspecies lateral gene transfer. The presence of resistance genes across the two geographic regions tested within one country supports the need for a careful, tailored, implementation and monitoring of antimicrobial stewardship.

## Introduction

Mastitis poses a risk to public health, animal welfare, and farm profitabilty worldwide^1–5^. Increased costs related to clinical mastitis are the result of reduced milk production and price, discarded milk, animal culling, mortality, labour and herd veterinary costs^5^. Both contagious and environmental pathogens have been implicated in intramammary infections and bovine mastitis. *Streptococcus uberis* is one of the most frequently identified environmental pathogens responsible for mastitis in dairy herds, a trend which appears to be increasing worldwide^4–6^. Mastitis control programs have been effective in decreasing the prevalence of contagious pathogens in well managed dairy herds^7^, but *S. uberis* remains responsible for a significant proportion of both clinical and subclinical intramammary infections^8^. In Australia, *S. uberis* associated mastitis accounts for one of every three cases of clinical mastitis^9^. This pathogen can be found in a variety of sites in the dairy cow environment such as bedding materials and milking equipment, as well as on other items within the dairy cows’ daily living spaces^10^. However, *S. uberis* is not only an environmental risk, this pathogen has also been associated with cow-to-cow transmission consistent with a contagious pathogen role for *S.uberis*^10–12^ and indicative of the complexity of the bacteria, environment and dairy cow relationship.

A number of different DNA-based techniques have been used to investigate the genetic diversity of *S. uberis* isolated from cases of bovine mastitis, including MLST^11, 13–16^. While MLST has provided insights into the population diversity of *S. uberis*^11, 16, 17^, it is limited in its ability to delve deeply into genetic variation of isolates. The recent availability of low-cost Whole Genome Sequencing (WGS) technology and advances in computational biology has made it possible to compare the entirety of the *S. uberis* genome and completely characterise the levels of heterogeneity between strains^18^.

WGS analysis of the genomic sequence of *S.uberis* strains has revealed both a capacity for nutritional flexibility and a large variety of metabolic capabilities that promote bacterial survival in different environments^18^, potentially responsible for the high degree of genetic diversity of *S. uberis* within the dairy herd environments^16, 19, 20^. Variations in genetic profile among *S. uberis* strains were not only observed in isolates originating from different dairy herds, but also in the pathogens present within the same herd^7, 21^. This inherently high genetic diversity renders the attribution of a specific set of virulence factors (and associated pathogenesis) to a given *S. uberis* strain/s challenging^12, 22, 23^. The development of strategies to control this important mastitis-causing organism necessitates monitoring of the genetic diversity and determination of the significance of any detected diversity to the disease pathogenesis^22, 24^. Therefore, the main objectives of this study were to compare *S. uberis* isolates cultured from dairy cows with clinical mastitis in distinctly different geographic and climatic regions of Australia. We then identified antibiotic resistance genes, virulence factors, and mobile genetic elements which could then inform antimicrobial stewardship programs. A secondary objective was to examine the diversity of the isolates, in particular their specific antimicrobial resistance profiles from these different regions to identify geographic or climatic associations that may exist as a potential basis for development of more effective and sustainable mastitis treatments for dairy cows.

## Results

### *S. uberis* mastitis isolates, sequence types and phylogenetic relationships

**Figure 1:**
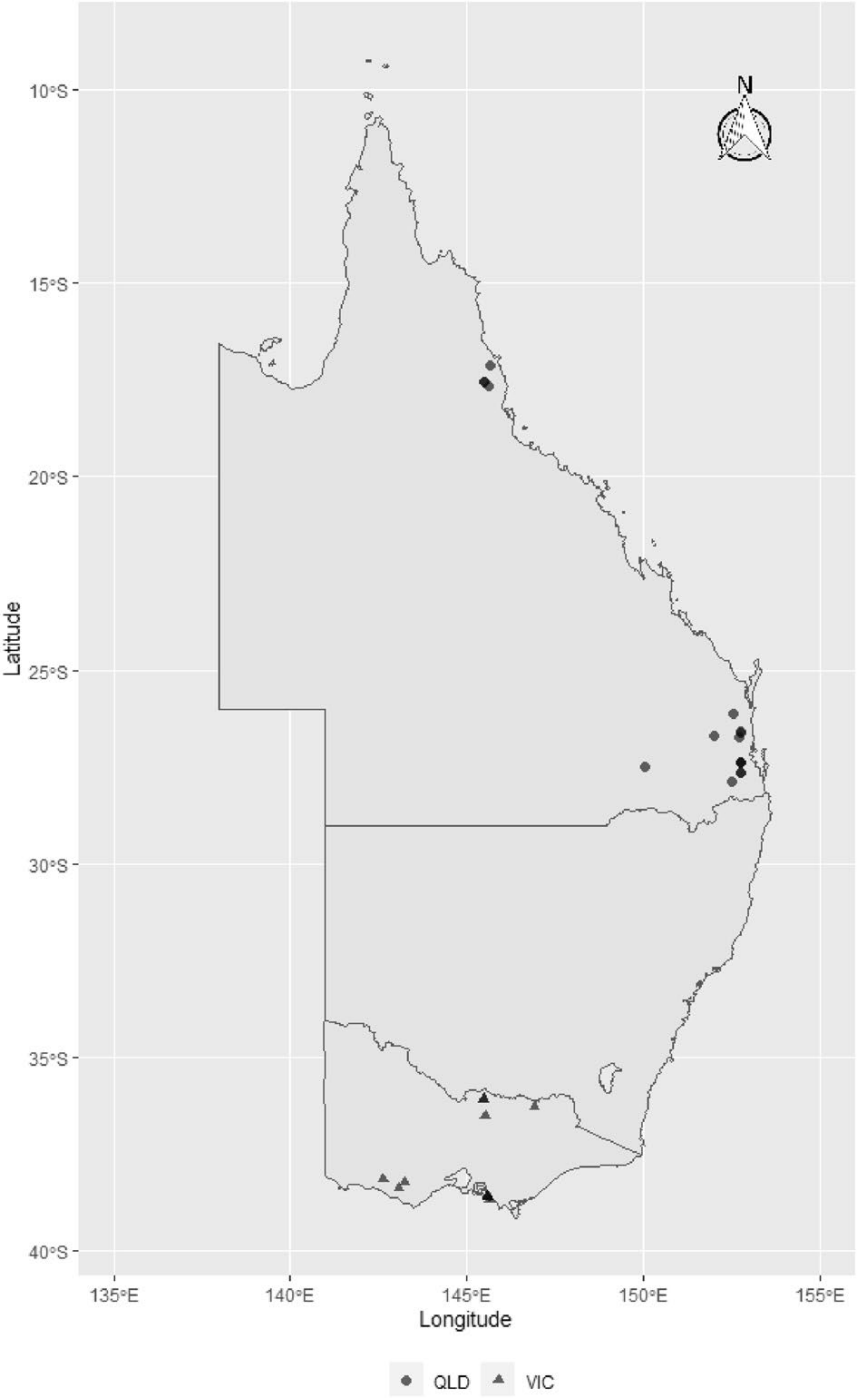
Map showing the geographical distribution of *S. uberis* isolates sourced from farms in climatically distinct regions across Australia: Queensland (QLD) farms (North QLD (circles), South QLD (circles)), and Victorian farms (triangles). The symbols represent the townships centroids were the farms are located. The map was generated using ozmap package version 0.3.6 [https://CRAN.R-project.org/package=ozmaps]^99^ implemented within R version 4.0.2 [https://www.R-project.org/]^100^).

**Figure 2:**
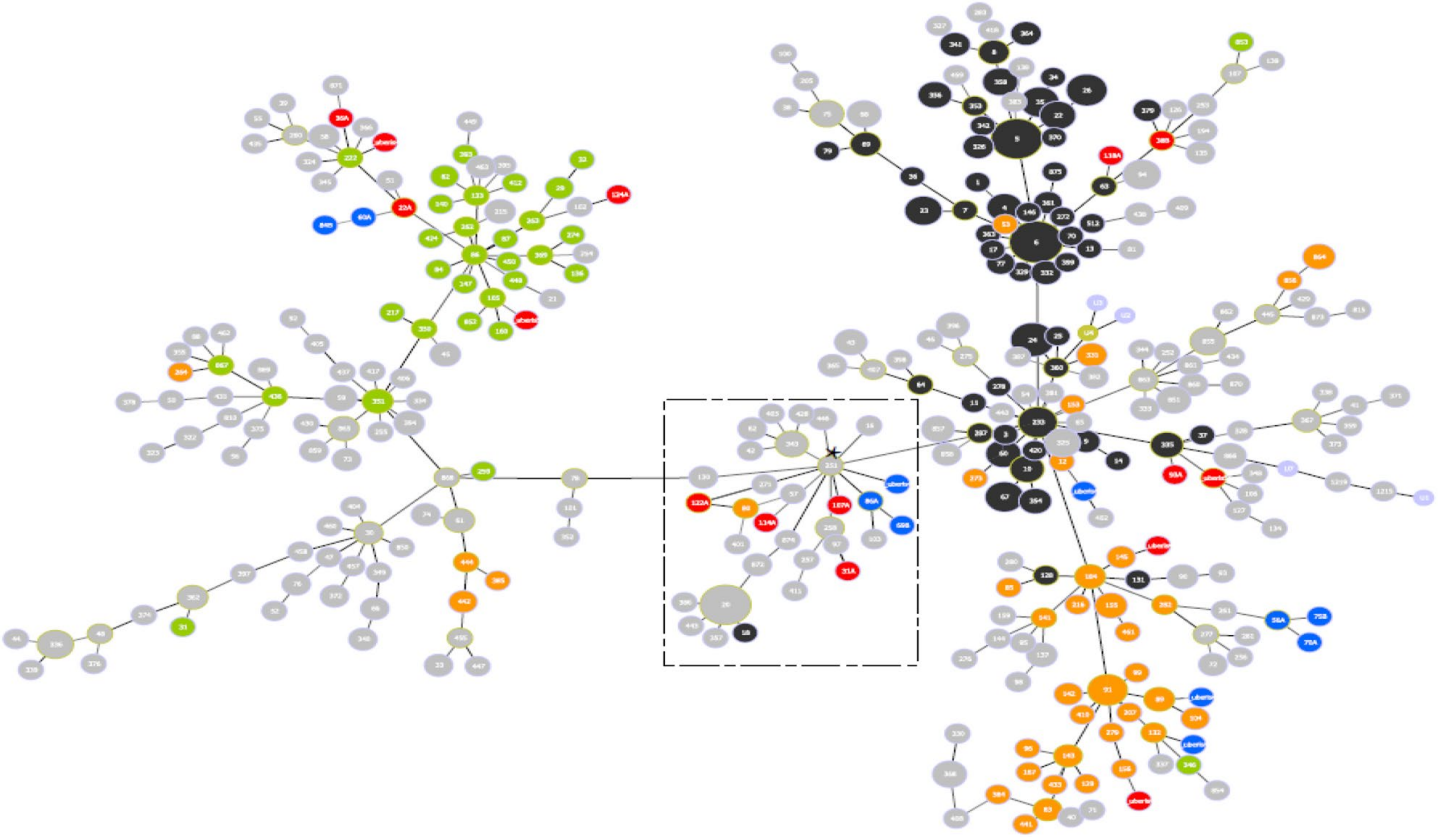
Multilocus sequence type (MLST)-based minimal spanning tree of 1220 *Streptococcus uberis* isolated from bovine mastitis milk as per NCBI database^103^ (as of 8 June 2020) to date. Coloured circles are Australian isolates from bovine origin (n = 240). The tree was calculated and generated using the goeBURST version 1.2.1 (http://www.phyloviz.net/goeburst/)^113^ full MST algorithm in Phyloviz version 2.0 (www.phyloviz.net)^112^. Node sizes reflect the number of isolates with specific MLST profile. Numbers within the nodes indicate the ST. Node colours refer to types of Global Clonal Complexes (GCC): GCC Strain Type (ST) 86 (green; left side of the plot), GCC ST143 (orange, right-bottom side of the plot), GCC ST5 (black, right-top side of the plot), isolates from Queensland are in red colour and Victorian isolates are in blue. Dashed rectangle show the location of the seven isolates to their possible ST founder ST251 (asterisk).

Eleven isolates from 10 locations in Victoria (VIC) and 16 isolates from 8 sites in Queensland (QLD) for a total of 27 isolates were collected and analysed in this study (Fig. 1). The 27 *S. uberis* genomes ranged in size from 1,832,555 to 2,019,389 bp (Table 1). There were 21 MLST^16^ sequence types (STs) represented within this collection. All sequence types (STs) were considered novel as they matched no known sequence types in the *S. uberis* MLST database (pubmlst.org/suberis)^25^. The closest ST matches were ST ~ 1223 (4/27) followed by ST ~ 222 (3/27). These were only present in QLD farms (Table 1). Analysis of GCC STs showed the most abundant STs to be GCC ST86 (8/27) and GCC ST143 (8/27) with GCC ST5 representing 4/27 isolates. All GCC ST86 isolates were from herds with high bulk milk somatic cell counts (270–400 × 10^3^ cells/mL). GCC ST143 isolates were predominantly (*p* = 0.048) found in Victoria (75%) while GCC ST86 isolates were predominantly found in Queensland (88%). Seven isolates (27%) did not belong to a defined GCC. Based on the goeBURST analysis, a possible founder ST for those 7 isolates was ST251. The relationship between the STs of this study and those reported globally in the *S. uberis* MLST database (pubmlst.org/suberis) is depicted in Fig. 2.

**Table 1:** Summary of *S. uberis* strains identified in this study, isolated from cows affected by bovine mastitis. MLST STs are also shown.

Isolate	Genome size (bp)	Source	State	MLST ST	GCC ST
*S. uberis* 22A	1,938,913	QLD1	Queensland	~ 222	86
*S. uberis* 31A	1,900,760	QLD2	Queensland	~ 97	–
*S. uberis* 36A	1,914,697	QLD3	Queensland	~ 222	86
*S. uberis* 38B	1,958,229	QLD3	Queensland	~ 253	5
*S. uberis* 42	1,883,527	VIC1	Victoria	~ 271	143
*S. uberis* 43	1,925,541	VIC2	Victoria	~ 402	5
*S. uberis* 44	1,879,665	VIC3	Victoria	~ 89	143
*S. uberis* 45	1,961,274	VIC4	Victoria	~ 190	–
*S. uberis* 46	1,899,378	VIC5	Victoria	~ 132	143
*S. uberis* 47	1,971,757	QLD4	Queensland	~ 27	143
*S. uberis* 48	1,927,709	QLD5	Queensland	~ 222	86
*S. uberis* 49	2,019,389	QLD6	Queensland	~ 145	143
*S. uberis* 50	1,938,127	QLD7	Queensland	~ 425	86
*S. uberis* 51	1,984,097	QLD8	Queensland	~ 56	86
*S. uberis* 56A	1,906,743	VIC6	Victoria	~ 261	143
*S. uberis* 60A	1,967,409	VIC6	Victoria	~ 412	86
*S. uberis* 69B	1,926,363	VIC7	Victoria	~ 271	–
*S. uberis* 70A	1,919,457	VIC8	Victoria	~ 256	143
*S. uberis* 75B	1,844,929	VIC9	Victoria	~ 156	143
*S. uberis* 86A	1,977,432	VIC10	Victoria	~ 90	–
*S. uberis* 93A	1,856,167	QLD9	Queensland	~ 67	5
*S. uberis* 106A	1,832,555	QLD6	Queensland	~ 1223	86
*S. uberis* 107A	1,832,592	QLD6	Queensland	~ 1223	–
*S. uberis* 114A	1,927,632	QLD7	Queensland	~ 1223	–
*S. uberis* 118A	1,894,094	QLD8	Queensland	~ 850	5
*S. uberis* 122A	1,886,974	QLD8	Queensland	~ 80	–
*S. uberis* 124A	1,909,330	QLD8	Queensland	~ 1223	86

~ indicates closest ST. All STs were considered novel. The isolation sources have been given codes for privacy reasons.

### Pangenome analysis

Pangenome analysis showed a total gene pool of 3764 genes (including RNA encoding), with the core genome making up 1509 genes (Table 2). The soft-core genome, found in ≥ 26/27 isolates, was made up of 1542 genes. Of these, 1380 genes (89.49%) could be assigned a Gene Ontology (GO) identity and descriptors (Data S5). The most abundant class of genes, based on predicted localisation of the encoded proteins was as ‘integral member of the membrane’ followed by ‘cytoplasm location’. Most genes appeared to be housekeeping. In addition, 64 encoded proteins predicted to be associated with proteolysis, 5 associated with virulence, 4 with adhesion, 1 with capsule polysaccharide biosynthetic process, and 1 was pillus-associated. Of the 5 virulence-associated genes, 3 encoded proteins that were predicted to be secreted: pauA, LytR, and the YSIRK-type signal peptide-containing protein. Several proteins associated with nutrients commonly found in abundance within the mammary tissue of dairy cows were also identified including fibrinogen binding proteins (WP_046389274.1, WP_012658173.1) and lactose catabolism proteins (*lacA*, WP_000215993.1; *lacB*, WP_000686149.1).

**Table 2:** Pangenome analysis results. The total number of genes and percent of total pangenome is shown.

Pangenome breakdown	Number of isolates^a^	Number of genes	Percentage of pangenome (%)
Core genes	27	1509	40.09
Soft core genes	26	33	0.88
Shell genes	5–25	534	14.19
Cloud genes	1–4	1688	44.85
Total genes	1–27	3764	100

^a^Number of isolates out of the total (*n* = 27).

### Virulence factors

To address the lack of traditional *S. uberis* virulence factors detected from the Virulence Factor Database (VFDB), a custom *S. uberis* virulence database was constructed, named the *S. uberis* Putative Virulence Database; _*Su*_PVDB (Data S2). This was built based on three main sources. Firstly, identification of translated homologues from the VFDB using relaxed alignment statistics (> 70% query cover and > 60% similarity) (Data S3). Secondly, experimentally-confirmed virulence factors identified from a number of publications^26–41^. Finally, inclusion of genes with virulence-associated GO descriptors (Data S5). Despite HasA^42^, PauA^43^, and PauB^39^ being non-essential for bovine mastitis, these three were included in the search for the sake of completeness. MtsB and regulator ScaR were also included as potential virulence factors because they were associated with the MtuA operon. To identify homologous virulence factors in *S. uberis*, the *Streptococcal* VFDB amino acid sequences were screened against the translated pangenome. Any sequences with > 70% query cover and > 60% similarity were considered putative homologues. Twenty-eight previously undescribed putative virulence factors were identified in *S. uberis* based on homology to known virulence factors in other *Streptococcal* species (Data S3). This custom database consisted of 53 total sequences (Data S2), which were screened against each isolate, as well as all 40 *S. uberis* isolates present within GenBank (Data S6).

The GO virulence-associated genes encoded an enolase/phosphopyruvate hydratase (WP_012658173.1), LytR family regulatory protein (SQG46876.1), YSIRK-type signal peptide-containing protein (WP_154591130.1), PauA a plasminogen activator A (WP_046388868.1), and a HU family DNA-binding protein, HlpA (WP_012658741.1). 90 soft core proteins were predicted to be secreted including PauA, LytR, and the YSIRK-type signal peptide-containing protein were all predicted to be secreted, along with pneumococcal-type histidine triad protein (Pht).

The average number of virulence factors per isolate was 27.7 factors (± Standard deviation [σ] = 2.6) from the current study. When the publicly available *S. uberis* genomes were screened, an average of 27.86 ± 2.14 σ putative virulence factors were identified from isolates associated with bovine mastitis (Data S6). The 3 human mastitis isolates also contained a similar amount, 27.67 ± 4.04 σ. The aquatic *S. uberis* isolate CAIM 1894 (SAMN03093229) contained a total of 3 putative virulence factors.

In the current study, the number of virulence factors per isolate ranged from 35 in *S. uberis* 36A to 24 in *S. uberis* 124A. Twenty-two putative virulence factors were found in every isolate. These factors included *biofilm putative glycosyltransferase, biofilm putative glycosyltransferase 2, cpsB, cpsC, cpsD/cps4D, cylA, fabG 1/cylG, fbpS, gtaB/hasC, hasC homologue gpsA, lmb, mga, mtuA, oppF, pauA, putative surface-anchored protein, rqcH/fbp54, scaR, scpA, sua, tagU 3/cps4A* and *srtA* (Fig. 4, Data S4). Moreover, there was a tendency for virulence factors to cluster by state (*p* = 0.09). No clustering was observed between isolation source and virulence factors. The accessory virulome was made up of putative virulence factors not present in every isolate. They consisted of 24 sequences (Fig. 4), with individual isolates containing the majority of them. *S. uberis* 56A was the only isolate to contain the putative virulence factors *cpsL* and *neuABCD* (*epsM/neuD, legI/neuB, neuA_1, neuC*).

### Putative sortase substrates, antimicrobial resistance and mobile genetic elements

*Streptococcal* sortase *srtA* has been shown to be essential for bovine mastitis^26^ and a previous study identified 9 proteins attached to the cell wall of *S. uberis* which were likely virulence-associated genes^44^. This enzyme cleaves and processes proteins containing particular motifs to allow external cell wall-anchoring. To confirm the importance of cell wall-anchored proteins, isolates were screened for the *srtA* gene, which had two variants with 85.9% similarity between them. 22/27 strains contained one *srtA* variant (WP_046390884.1) while 5/27 strains contained another (WP_037592200.1). This was the result of the 90% identity cut-off criteria used in the pangenome analysis. Within this constraint, sortase *srtA* was still considered a core gene.

To analyse SrtA substrates, core protein-coding sequences were screened for several motifs including LPXTG^44, 45^, LPXXXD^44^, LPXTA^46, 47^, QVPTGV and LPSTGE^48^. 12 protein-coding sequences were identified when excluding the more variable LPXXXD motif, and 102 including it. As only core genes were screened, there was an increased likelihood of finding essential virulence factors. Some putative sortase substrates of note included a transmembrane protein containing a xanthine permease domain (WP_046392124.1), 7 coding sequences (CDS) associated with the bacterial global response to DNA damage (SOS), peroxiredoxin *ahpC* (WP_015912043.1), and transmembrane branched-chain amino acid transport protein (WP_046393325.1) (Data S5).

Overall, antibiotic resistance genes were not commonly found within the genomes. Only *S. uberis* 45, 47, 48 and 51 contained resistance genes and most of these were predicted to encode resistance to lincosamides, due to presence of *lnuC* or *lnuD* (Table 3). Antibiotic resistance genes were found in both Victorian and Queensland isolates and in a number of STs. Screening NCBI for *LnuC* revealed it was not identified in any *S. uberis* isolates in the database. A multidrug antibiotic resistance cluster was identified in both *S. uberis* 48 and 51. The cluster consisted of *mel*/*mef*(*A*), *mrsE*, *vatD* and *lnuD*. Despite *S. uberis* 47 containing *lnuD*, it did not contain the cluster. The two isolates containing the cluster were found in different isolation sources within Queensland. They both belonged to GCC ST86. The gene cluster is associated with bacterial resistance to lincosamides, macrolides, oxazolidinones, phenicols, pleuromutilins, streptogramins and tetracyclines.

**Table 3:** Table showing antibiotic resistance profiles of *S. uberis* isolates in this study.

Isolate/resistance gene	Lincosamides	Streptogramins	Macrolides, oxazolidinones, phenicols, pleuromutilins, streptogramins, tetracyclines
*S. uberis* 45	*lnuD*		
*S. uberis* 47	*lnuC*		
*S. uberis* 48	*lnuD, mel*, *mrsE*,	*vatD*	*mel, mrsE*
*S. uberis* 51	*lnuD, mel*, *mrsE*,	*vatD*	*mel, mrsE*

The antibiotic class is shown along the x-axis, and the isolate is shown along the y-axis. The gene responsible for resistance is shown.

One-third (9/27) of the isolates contained putatively intact bacteriophages (Table 4). *S. uberis* 38B contained two bacteriophages. The *Streptococcus* prophage 315.2 was found in isolates *S. uberis* 38B and *S. uberis* 47, both Queensland isolates. There were no virulence genes associated with any bacteriophages. Transposons and insertion sequences were identified in 21/27 isolates (77.78%) (Table 5). None were associated with virulence factors. No plasmids were found in the isolates.

**Table 4:** Table of putatively intact bacteriophage presence within the *S. uberis* isolates identified in this study.

Isolate	Region length (kb)	Closest bacteriophage hit	Accession of closest hit
*S. uberis* 38B	36.8	*Streptococcus* phage SMP	NC_008721
*S. uberis* 38B	42.2	*Streptococcus* prophage 315.2	NC_004585
*S. uberis* 47	43.9	*Streptococcus* prophage 315.2	NC_004585
*S. uberis* 49	40.8	*Enterococcus* phage phiFL4A	NC_013644
*S. uberis* 50	55.5	*Streptococcus* phage 5093	NC_012753
*S. uberis* 60A	59.5	*Streptococcus* phage PH10	NC_012756
*S. uberis* 70A	36.4	*Streptococcus* phage T12	NC_028700
*S. uberis* 93A	36.7	*Streptococcus* phage 315.3	NC_004586
*S. uberis* 118A	42.8	*Streptococcus* phage P9	NC_009819
*S. uberis* 122A	41.9	*Streptococcus* phage T12	NC_028700

The closest-related phage are also shown, along with the accession numbers.

**Table 5:** Transposon and insertion sequences within the *S. uberis* isolates identified in this study.

IS family	Isolates	Total
IS256	*S. uberis* 46	1
IS3	*S. uberis* 106A; *S. uberis* 107A; *S. uberis* 114A; *S. uberis* 122A; *S. uberis* 122A; *S. uberis* 124A; *S. uberis* 22A; *S. uberis* 38B; *S. uberis* 43; *S. uberis* 44; *S. uberis* 47; *S. uberis* 49; *S. uberis* 51; *S. uberis* 56A; *S. uberis* 70A; *S. uberis* 75B; *S. uberis* 86A	19
IS30	*S. uberis* 118A; *S. uberis* 42; *S. uberis* 44; *S. uberis* 56A; *S. uberis* 69B; *S. uberis* 70A; *S. uberis* 75B	7
IS6	*S. uberis* 31A	1
ISL3	*S. uberis* 86A	1
Novel	*S. uberis* 22A	1

Isolates which contain IS families are separated by semicolons. *S. uberis* 122A contains two different IS3 regions and is included twice in this table.

## Discussion

**Figure 3:**
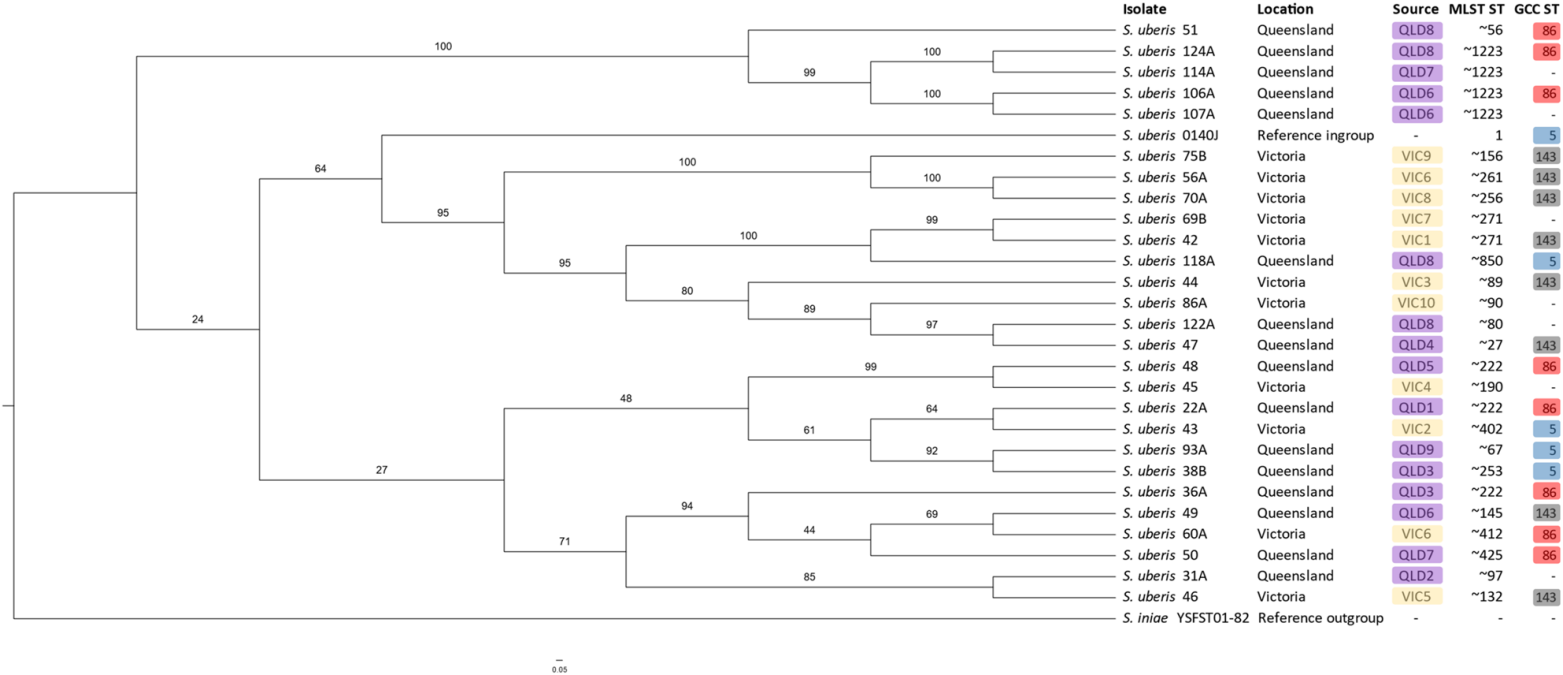
Maximum likelihood phylogenetic tree based on core genome alignment between the *S. uberis* isolates identified in this study. *S. iniae* YSFST01-82 (accession number: GCF_000831485.1) was used as an outgroup and *S. uberis* 0140J (accession number: AM946015) (MLST ST1, GCC ST5) as an ingroup. Bootstrap values are shown in numbers along branches. Purple labels show Queensland isolates. Yellow labels show Victorian isolates. MLST STs are shown, as well as GCC STs. GCCs are coloured, GCC ST86 coloured red, GCC ST 5 coloured blue, GCC ST143 coloured grey. Core genome alignment was performed in roary (version 3.13.0)^115^ and maximum likelihood tree was built in RAxML version 8.2.10 (raxmlHPC-PTHREADS-SSE3)^120^ with 1000 replicates. Tree visualised in FigTree version 1.4.4 (http://tree.bio.ed.ac.uk/software/figtree/). For figure clarity, the phylogenetic tree was visualised using a cladogram transformation. Data S1 shows the untransformed tree, along with an isolate-only tree which shows very high bootstrap support at almost every node (~ 100%) due to the increased number of genes in the core genome alignment.

**Figure 4:**
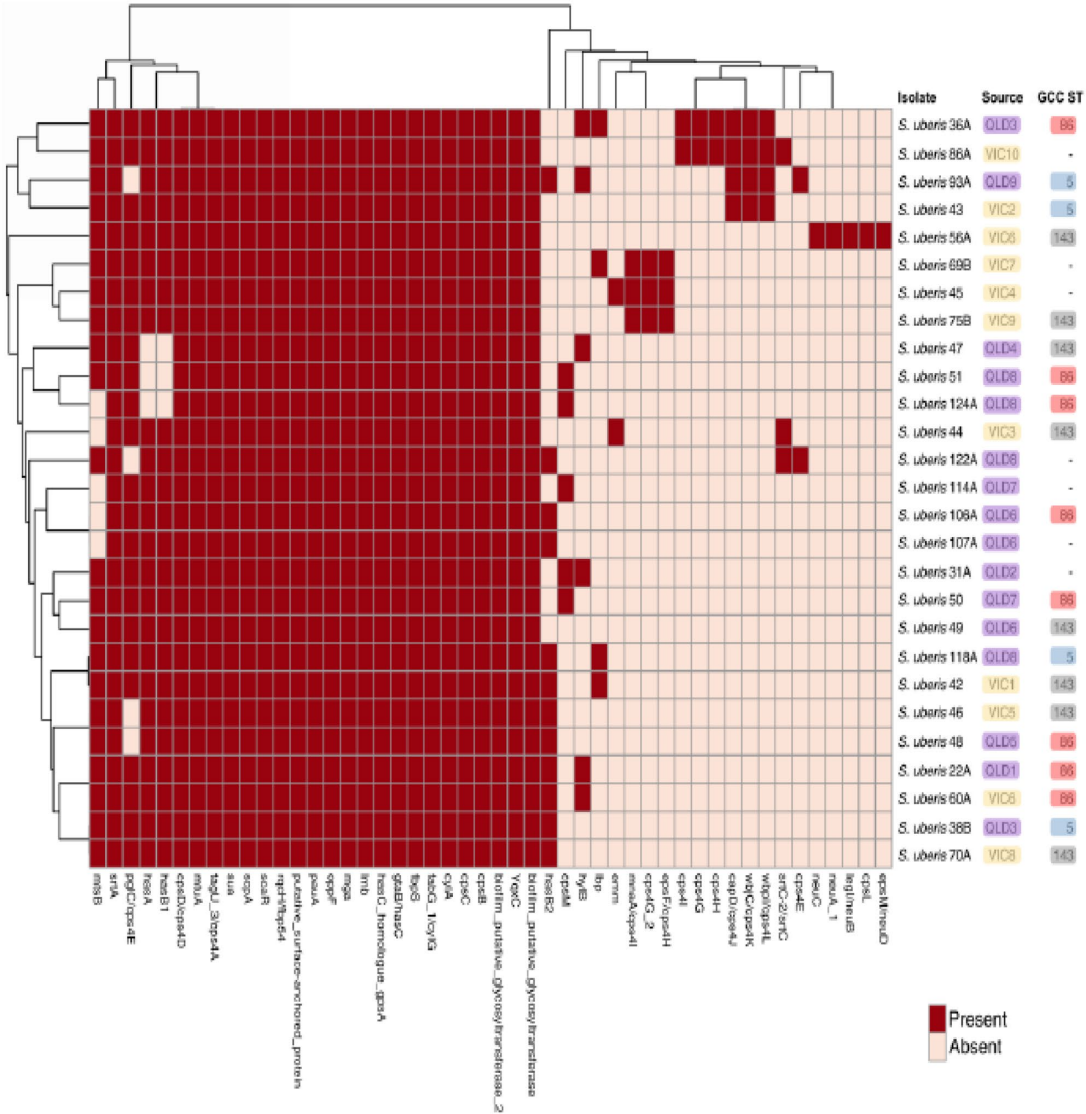
Heatmap showing the presence/absence of virulence and putative virulence genes (x-axis) within the *S. uberis* isolates identified in this study (y-axis). Presence of virulence genes shown in red, absence in light pink. Core virulence genes are found in every isolate (27/27), while accessory virulence genes are found in < 27 isolates. Victorian isolates are coloured in yellow, Queensland in purple. GCCs are coloured, GCC ST86 coloured red, GCC ST5 coloured blue and GCC ST143 coloured grey. Data generated using ABRicate version 1.0.1 (https://github.com/tseemann/abricate)^121^ with the VFDB^122^ and the custom _*Su*_PVDB (Data S2). Heatmap produced using ClustVis (https://biit.cs.ut.ee/clustvis/)^124^ and Inkscape version 0.92 (https://www.inkscape.org).

*Twenty-nine* herds located in three climatically distinct geographic regions of Australia were screened and from them 27 independent *S. uberis* isolates were obtained and their genetic relatedness was assessed. The diverse range of MLSTs observed in this study is consistent with previous surveys of bovine mastitis-associated *S. uberis* in that there was a large variety of STs identified^49^. In one study, 33 MLST STs were identified in their group of 46 isolates, the most common STs being MLST ST60 and ST155^49^. In the current study, none of the MLST STs in the isolates were identical, although some were similar such as MLST ST ~ 1223. However, GCC STs were more consistently represented (Table 1) with three GCC STs (ST86, ST143, GCC ST5) representing 20 of the 27 isolates. This finding is mostly consistent with the published literature that has identified GCC ST143 and GCC ST5 as the most common GCCs found in *S. uberis* isolates obtained from Australian cases of clinical mastitis^11, 16, 17, 23^. The observation that GCCs clustered by region was notable, given that GCC ST86 isolates were more prevalent in QLD compared to Victoria, while GCC ST143 isolates were more prevalent in Victoria. This study provides observational evidence that *S. uberis* GCC ST86 isolates, which had been previously thought to lack pathogenicity required to cause clinical mastitis^49^, can contribute to disease. Further investigations such as experimental animal trials will be required to confirm this observation. If GCC ST86 isolates were indeed responsible for clinical mastitis, it would also be consistent with the lack of strong phylogenetic clustering of GCC ST86 (Fig. 3); the putative virulence factor profiles which showed consistent differences between GCCs (Fig. 4). Isolation of *S. uberis* GCC ST86 strains from cows afflicted with bovine mastitis may be indicative of a shift in pathogenic lineages within the Australian *S. uberis* population. Alternatively, this provides evidence that sequence typing alone is not a sufficiently granular approach to associate with pathogenicity of *S. uberis* in the bovine mammary gland, neither the core genome-based phylogenetic clustering (Fig. 3) nor the putative virulence factor profiles (Fig. 4) align closely with GCC typing in parallel. Our results indicate that all isolates likely possess virulence factors promoting invasion of host tissue, survival in the host environment, evasion of the host immune response, and internalization in the mammary gland cells^49^.

These observations indicate that the relative importance of an environmental source and possible cow-to-cow transmission in the development and risk of mastitis also depend on geographical factors, and the transmissibility and disease potential of the particular strains circulating in the cow’s environment^10, 20, 50^. It could also impact on the relative success rate of treatment protocols^51^ and management practices and the apparent ‘success’ and ‘failure’ rates in different regions or on different properties. At present, attention to possible environmental sources of infection, good standards of milking hygiene, and proper treatment of *S. uberis* mastitis remain important measures in attempts to control transmission of *S. uberis*^20, 23^.

A maximum likelihood phylogeny of the core genome demonstrated the clustering of MLST STs and indicated that the scheme was, at times, suitable (Fig. 3) for *S. uberis.* For example, MLST ST ~ 1223 clustered well, however MLST ST ~ 222 did not. Given the amount of novel STs identified (100% of isolates), the usefulness of the ST was of limited value to describing the evolutionary lineage of this highly recombinant species^52^. While the GCCs clustered with other bovine mastitis-associated *S. uberis* (Fig. 2), the inconsistent phylogenetic clustering between GCC STs (Fig. 3) meant that these GCCs did not appear to accurately capture the assumed evolutionary lineages of the bovine mastitis-associated *S. uberis* isolates identified in this study. Given the reducing cost of whole genome sequencing and greater power for genomic and phylogenetic analysis compared with the multiple sanger sequencing reactions required for MLST and GCC ST analysis, it would be appropriate to rely more on higher resolution WGS in future diagnostics. There appeared to be two clades containing state-only isolates: a Queensland clade including *S. uberis* 51, 124A, 114A, 106A and 107A, which almost all shared the same MLST ST (~ 1223) and GCC ST (86); and a Victorian clade containing *S. uberis* 75B, 56A, and 70A, in which almost all sharing the same GCC ST (143). Within these two specific clades, it is likely that the isolates diverged from a common lineage within their respective states. Every other identified clade appeared to contain a mixture of Victorian and Queensland isolates. Phylogenetic clustering within a source point was not observed in many cases, such as with QLD7, QLD8, QLD9 and VIC6 isolates. This observed divergence was much greater than originally expected. The most likely explanation of this finding is environmental acquisition of novel *S. uberis* bovine mastitis-associated isolates^23, 41, 50^, rather than an acquisition of monophyletic descendants such as would be expected with a contagious pathogen. Given this observation, further analysis of environmental *S. uberis* reservoirs is recommended.

The pan and core genome genes were consistent with previous pangenome analyses, showing a core genome of 1550 genes using 13 clinical and sub-clinical bovine mastitis isolates^27^ and ~ 1530 genes using 21 bovine mastitis isolates^52^. Core genes associated with nutrients found in abundance within the mammary tissue of cows, including two fibrinogen binding proteins, were identified (WP_046389274.1, WP_012658173.1). This is a notable finding given the fibrinogen concentration in the milk of cows affected by mastitis is significantly higher than that of healthy cows^53^. Two genes associated with lactose catabolism were identified, *lacA* (WP_000215993.1) and *lacB* (WP_000686149.1), which is also notable given the abundance of lactose in bovine milk. This may explain the presence or adaptation of *S. uberis* to mammary tissue.

As strain specific pathogenicity has been observed with *S. uberis*^54^, this indicates bacterial pathogenic factors are involved, not host-specific factors. Notably, the isolates from this study shared similar numbers of putative virulence factors as the publicly available bovine mastitis isolates, as well as human mastitis isolates (Fig. 4, Data S4, Data S6). This indicates a common set of virulence determinants responsible for cross-host mastitis pathogenesis.

The core virulence factors identified in every isolate (Fig. 4, Data S4) may partially explain the pathogenic behaviour of *S. uberis*. There were several core genes of note including *cpsBCD* and *cps4a* which encode products involved in capsular polysaccharide formation^40^, as-well-as, two putative glycosyltransferases involved with biofilm formation^38^. *cylAG* were identified, which contribute to hemolytic activity^55^, corresponding to an ABC transporter and an enoyl reductase involved in fatty acid biosynthesis^56^. This analysis confirmed *hasA* as a non-essential virulence factor, as it was only found in 24/27 isolates, and also confirmed *mtuA* as an essential virulence factor^33^, as it was found in all isolates. *rqcH*/*fbp54*, a fibronectin-binding protein and putative fibronectin-binding protein *fbpS* were also found in all isolates. This has been shown to be important for adhesion and a key virulence factor in *Streptococcus pyogenes*^57^, as-well-as, in *Streptococcus suis*, but not essential for virulence^58^. Also found in every isolate was *scpA*, a C5a peptidase, which has been shown to delay accumulation of leukocytes attracted to *S. pyogenes* by cleaving the serum chemotaxin C5a, indicating it is an essential virulence factor^59^. *hasC* was also found in all isolates; it is essential for hyaluronic acid capsule production^29^. Upon loss of this gene, *S. uberis* becomes susceptible to phagocytosis by bovine neutrophils, indicating it is an important virulence factor^29^. *lmb*, encoding a laminin binding protein important for bacterial colonisation^37^ was also found in every isolate. *Mga*, a virulence regulator has been shown to be important for a number of phenotypes including biofilm formation, growth in whole human blood and soft tissue and phagocytosis resistance^60, 61^. This was also identified in all isolates of *S. uberis* in this study. *sua*, an adhesin, has been experimentally verified in *S. uberis* isolates from dairy cows and identified as a conserved gene^62^. Our study confirms this as it is conserved in all isolates. *oppF*, an oligopeptide permease, shown to be important for auxotrophic amino acid acquisition from bovine milk^34^, was also found in every isolate. *scaR*, another virulence regulator which upregulates during low levels of Mn^2+^^63^ was also found in every isolate. Given that manganese is present in cow’s milk at 0.02–0.05 µg/mL^64^, it is likely *scaR* upregulates virulence-associated genes in response to mastitis infection. Finally, a putative surface anchored protein was also found in all isolates. This was experimentally identified as a sortase substrate, anchored to the surface in *S. uberis*^44^. It contains a G5 domain, which is involved with N-acetylglucosamine binding and is found in a number of different enzymes, ranging from biofilm proteins to IgA-cleaving peptidases^65^. The *neuABCD* locus is involved in sialic acid synthesis and their presence in *S. uberis* 56A indicates it may produce a capsule to assist with immune system evasion^66, 67^.

Other core virulence genes included enolase/phosphopyruvate hydratase (WP_012658173.1), involved in plasminogen and adhesion in *Streptococcal* species^68^ and LytR family regulatory protein (SQG46876.1) essential for attaching capsules and teichoic acids to cell wall^69^. Also a YSIRK-type signal peptide-containing protein (WP_154591130.1), *PauA* a plasminogen activator A (WP_046388868.1) which has been already described as a non-essential virulence factor in *S. uberis*-related bovine mastitis^43^. *Pht*, a putatively secreted gene has been implicated in adhesion to epithelium in *Streptococcus pneumonia*^70^. Finally, a HU family DNA-binding protein, *HlpA* (WP_012658741.1) has been shown to bind to double-stranded DNA involved in repair and recombination^71^ in times of cellular stress^72^ and is involved in tissue inflammation of *Streptococcal* species^73^.

Scanning the core genome for putative sortase substrates revealed several genes which may also explain behaviour of *S. uberis*, including a transmembrane branched-chain amino acid transport protein (WP_046393325.1), which has been shown to transport leucine, isoleucine and valine via proton motive force^74^, all three of which are found in excess within bovine milk^75^. Furthermore, bacterial loads of *Staphylococcus aureus*, another bovine mastitis-causing organism have been correlated with these amino acids in milk^76^.

Another putative *srtA* substrate of note is a transmembrane protein containing a xanthine permease domain (WP_046392124.1), which allows uptake of xanthine. Xanthine and hypoxanthine are commonly found in bovine milk^77^, along with bovine enzyme xanthine oxidoreductase. Bovine xanthine oxidoreductase catalyses oxidation of hypoxanthine to hydrogen peroxide^78^ and xanthine, and of xanthine to uric acid, among many other substrates^79^. Of note, is the demonstrated antimicrobial activity against Gram-positive and negative bacteria in milk via production of reactive oxygen/nitrogen species, namely hydrogen peroxide and nitric oxide in both humans and cows^78, 80^. All *S. uberis* isolates identified in this study containing an externally-located protein which competes with this precursor antimicrobial compound may indicate a novel virulence factor which allows *S. uberis* to colonise and infect the udder, causing bovine mastitis. The core genome contained 7 genes associated with the bacterial SOS response (Data S5), which may be involved in combating damage caused to DNA by reactive oxygen/nitrogen species^81^. Also found in the core genome was peroxiredoxin *ahpC* (WP_015912043.1), which breaks down hydrogen peroxide and has been shown in *Streptococcus agalactiae* to bind heme^82^.

We propose the xanthine permease domain protein uptakes free hypoxanthine and xanthine found in bovine milk. This likely competes with bovine enzyme xanthine oxidoreductase which usually converts xanthine to the antimicrobial, reactive oxygen species, hydrogen peroxide. At least 7 genes associated with the bacterial SOS response are likely involved in combating damage caused to DNA by reactive oxygen/nitrogen species. The presence of core genes such as these demonstrate adaptation of *S. uberis* to the bovine host, and as one facet of *S. uberis*-associated bovine mastitis. It is possible the genes highlighted here demonstrate the Red Queen hypothesis^83^ between host and pathogen^84, 85^, which can be summarised as follows: organisms living within a dynamic environment (bovine host) require matching rates of evolution to maintain colonisation. Co-evolution between host and parasite is a widely recognised consequence of natural selection^86^ and continually develops as genetic arms-race.

There were a limited number of antibiotic resistance genes found in this study. This finding is consistent with some previous dairy herd surveys^9^, but inconsistent with others that detected a broad and diverse group of antibiotic resistance genes^13, 87, 88^. Four *S. uberis* isolates found across Victoria and Queensland herds contained a combination of *InuC* and a multidrug resistance cluster including *mel/mef(A), mrsE, vatD and lnuD.* The presence of an antimicrobial resistance gene cluster not associated with any known insertion sequences or mobilizable elements indicates this may have been acquired through uptake and integration of free DNA. The two isolates that contain this cluster, *S. uberis* 48 and 51 are not directly phylogenetically related in the context of other isolates, which may indicate this chromosomal integration occurred independently. Further evidence of this can be seen in the variable genomic context of this cluster, which is not consistently placed within the genome (data not shown). The *mel*/*mef*(*A*) gene has been reported in *S. pneumoniae*^89^ and *S. pyogenes*^90^ but not in *S. uberis*. *S. uberis* was then screened via NCBI for *mel*/*mef*(*A*). Only one protein sequence was identified (accession: WP_046391446.1) from *S. uberis* 6780 (accession: JATD00000000), also isolated from milk from a cow udder afflicted with bovine mastitis^27^. This provided further evidence that this observation is a rare occurrence not associated with known mobilizable elements. The *mel*/*mef*(*A*) gene encodes resistance to lincosamides, macrolides, oxazolidinones, phenicols, pleuromutilins, streptogramins and tetracyclines^91^. The *msr* family *ABC-F* type ribosomal protection gene, which is homologous to the coding sequence of MrsE (99% query coverage, 79% similarity), encodes resistance to streptogramins, phenicols, pleuromutilins, lincosamides, oxazolidinones, tetracyclines, macrolides and other antibiotics which target the peptidyl-transferase region of the ribosomal subunit^92^. The next gene in the cluster encodes a hypothetical protein with no known function or domains. This is followed by *vatD*, a streptogramin A O-acetyltransferase resistance gene^93^. The final gene in the cluster is *lnuD*, encoding a nucleotidyltransferase, which has been shown to provide lincosamides resistance. The *lnuD* and non-cluster *lnuC* genes encode O-nucleotidyltransferases and inactivate lincosamides by adenylylation^94^. The *lnuD* has been previously reported in *S. uberis* isolated from clinical bovine mastitis^95^, but the *lnuC* gene has only been identified in human *S. agalactiae* isolates^96, 97^, suggesting recent acquisition by bovine mastitis-associated *S. uberis.* As *lnuC* is a transposon-mediated resistance gene^97^, this may represent the first observed interspecies jump into bovine mastitis-associated *S. uberis.* Lincosamide (lincomycin, clindamycin, and pirlimycin) usage and resistance should be carefully monitored now this mobilizable gene is within bovine mastitis-associated *S. uberis* populations. It would be of value to determine if a similar pattern exists in other streptococcal species present in the dairy farm environment. Further work is needed to verify these findings and determine antimicrobial gene reservoirs and the mechanism of transfer. A limitation of this study was the small number of herds and limited number of isolates cultured from clinical mastitis cases. However, herd (farm) type, breed of dairy cow, and herd size were representative of Australian herds. Nevertheless, the presence of these genes in some of the isolates indicates that antibiotic usage should be carefully tracked and monitored to produce and maintain an up-to-date understanding of the level of antimicrobial resistance within dairy populations in the country.

## Materials and methods

The study and all experimental procedures and protocols were approved by the Animal Ethics Committee at the University of Queensland (animal ethics approval numbers: SVS/ANRFA/540/18 and SVS/043/18/TERRAGEN), and all methods were performed in accordance with the relevant institutional guidelines and regulations. The methods described in the current study and reported results were compliant with ARRIVE guidelines for reporting animal research^98^.

### Sample collection

This study was part of a larger project conducted between March and June 2019 based on 430 milk samples collected from 29 dairy herds located in Queensland [QLD] (North Queensland, n = 9 herds; Southeast QLD, n = 9 herds), and Victoria ([VIC], n = 11 herds) (Fig. 1; map figure was generated using ozmap package version 0.3.6 [https://CRAN.R-project.org/package=ozmaps]^99^ implemented within R version 4.0.2 [https://www.R-project.org/]^100^). A 2-week rolling average of herd bulk somatic cell counts (BMTSCCs) was used to arbitrarily divide the herds into three categories (≤ 150 × 1000 cells/mL, > 150 to 300 × 1000 cells/mL, and > 300 × 1000 cells/mL). BMTSCCs categories were used as an a priori assumption for the risk of mastitis in the herd. Herd selection was based on ease of access to the farm location and the cooperation of the dairy farm owners and their associated veterinary practices.

Detailed methodology is described elsewhere^101^. Briefly, milk samples were collected from eligible dairy cows with a new case of clinical mastitis. Chronic mastitis cases (apparently healthy cow with lumps palpable in the udder, and mild changes to milk) and subclinical mastitis cases were not eligible for study enrolment. An enrolment eligible clinical mastitis case was defined as a previously apparently healthy lactating dairy cow of any age, breed, or stage of lactation that was experiencing a new case of clinical mastitis, defined as either the first occurrence of a mastitis event in the current lactation or a mastitis event occurring at least 21 days following a previous mastitis event that has clinically resolved or achieved a clinical cure^102^. Eligible cases must have not received systemic or intramammary antimicrobials, anti-inflammatory medications, or topical treatments in the 2 weeks prior to becoming a new case, nor immediately prior to sample collection. Milk samples were collected aseptically from individual quarters into separate sterile tubes, immediately capped and placed in a − 20 °C freezer. Collected samples were transported and delivered frozen to the Veterinary Laboratory Services of the University of Queensland for bacterial culture.

### *Streptococcus uberis* isolation

At the laboratory, milk samples were mixed thoroughly, 100 µL streaked onto Sheep Blood Agar (SBA, P2133 Sheep Blood Columbia Agar Plates, Thermofisher), and the SBA plate incubated aerobically at 37 °C for 18–24 h. *S. uberis* isolates were initially identified using conventional microbiology laboratory tests (Gram stain appearance and catalase production). Matrix-assisted laser desorption ionization-time of flight mass spectrometry (MALDI-TOF MS; Bruker Daltonik, Bremen, Germany) was used to confirm the identification. Individual colonies were sub-cultured on SBA plates and incubated aerobically at 37 °C for 18–24 h. Pure isolates were then incubated in 2 mL of Brain Heart Infusion (BHI) broth, subsequently mixed with 20% glycerol, and stored at − 80 °C.

### DNA extraction

Stored *S. uberis* isolates were batch thawed and cultured in BHI broth (37 °C, orbital shaker at 300 rpm, 18–24 h). Genomic DNA was extracted using DNeasy PowerFood Microbial Kit (QIAGEN Chadstone, Victoria, Australia) with minor modifications. Eight mL of culture liquid was centrifuged (15 min, 4 °C, 20,000×*g*) to pellet the bacteria. The pellet was then resuspended in 450 µL of lysis buffer and incubated for 10 min at 65 °C. Proteinase K (Proteinase K, QIAGEN Chadstone, Victoria, Australia) (25 µL) was added and incubation continued for an additional 20 min at 65 °C. This Proteinase K modification to the DNeasy protocol increased the final quantity of extracted DNA (data not shown). Thereafter, the whole component was transferred to a Powerbead tube, secured horizontally to a vortex adapter, and vortexed at maximum speed for 10 min. After washing to remove protein and other inhibitors, purified DNA was eluted and the cconcentration and purity of the isolated genomic DNA determined using a NanoDrop ND-1000 spectrophotometer. A sample of DNA was considered acceptable if the A260/280 ratio was ~ 1.8.

### Whole genome sequencing

The New England Biolabs NEBNext Ultra II FS DNA Library Prep Kit for Illumina for multiplexing was used according to the manufacturer’s instructions (New England Biolabs, Notting Hill, Victoria, Australia) to construct whole genome sequencing libraries. Libraries were sequenced (Illumina NextSeq 500 instrument, 300 cycle mid-output kit; 2 × 150 bp paired end) at the Centre of Health Genomics and Informatics, University of Calgary, Canada, to obtain an average coverage depth of > 100 and a read retention of > 98%. The draft genome sequences of the 27 *S. uberis* isolates have been deposited with GenBank^103^ under BioProject PRJNA669385.

### Whole genome assembly and annotation

Primer sequences were removed and reads were quality trimmed using cutAdapt^104^. The Nullarbor pipeline^105^ was used to process the samples. SKESA assembler (version 2.3.0)^106^ was used for de-novo assembly. Assemblies were annotated using prokka (version 1.14.5)^107^. Details about the parameters used in different packages can be found at https://github.com/bananabenana/genomics_methods_S.uberis/blob/main/commands_run.

### Multi-locus sequence typing

Assemblies were taxonomically assigned, analysed for MLST and were possible assigned a GCC, sequence types (STs)^108, 109^, and scored for genome distance using Type (Strain) Genome Server (TYGS) (https://tygs.dsmz.de)^110^ (date accessed: 19 May 2020). MLSTs were confirmed using mlst_check version 2.1.1706216^111^ and MLST databases accessed on 5 June 2020. Phyloviz version 2.0 (www.phyloviz.net)^112^ and the goeBURT algorithm^113^ were used to visualise the data. Pairwise comparison of genome sequences among the set of genomes were conducted by calculating precise distances using the Genome BLAST Distance Phylogeny approach (GBDP) under the algorithm 'coverage' and distance formula d5^114^. These distances were used to determine the genome similarities for each of the genome pairs. 100 distance replicates were calculated each. Digital DDH values and confidence intervals were calculated using the recommended settings of the GGDC 2.1^114^.

### Pangenome analysis

Pangenome analysis was performed using roary (version 3.13.0)^115^, splitting paralogues and setting the identity threshold at 90%. This list was then annotated and characterised via screening sequences against NCBI and Gene Ontology^116^ using the PANNZER2 server^117^. Signal sequences were obtained from the core genome using SignalP 5.0 (Linux x86_64)^118, 119^.

### Phylogenetic analysis

A core genome alignment generated by roary was performed, including an outgroup *S. iniae* YSFST01-82 (accession number: GCF_000831485.1) and in-group *S. uberis* 0140J (accession number: AM946015) ST1. RAxML (raxmlHPC-PTHREADS-SSE3 version 8.2.10)^120^ was used for maximum likelihood and rapid bootstrap analysis with 1000 replicates. The bipartitions output file was visualised in FigTree version 1.4.4 (http://tree.bio.ed.ac.uk/software/figtree/) and bootstraps visualised. Inkscape version 0.92 (https://www.inkscape.org) was used to prepare the figure.

### Identification of putative virulence factors

ABRicate version 1.0.1 (https://github.com/tseemann/abricate)^121^ was used with the VFDB^122^ and a custom gene list, the _*Su*_PVDB (Data S2). The custom gene list was based on sequences identified in the literature described to be *S. uberis* virulence factors with experimental or strong computational evidence^26–41^, as well identifying homologues of *Streptococcal* virulence factors. In addition to the isolates genomes reported in this current study, 40 additional publically available genomes (Data S6)^27, 123^ were retrieved from GenBank and screened for putative virulence factors. To identify homologous virulence factors in *S. uberis*, the *Streptococcal* VFDB amino acid sequences were screened against the translated pangenome. Any sequences with > 70% query cover and > 60% similarity were considered putative homologues (Data S3). The output was then visualised in ClustVis (https://biit.cs.ut.ee/clustvis/)^123^. No scaling was performed on the data and clustering was performed on rows and columns using the ‘Euclidean’ measure. To identify core, putative sortase A substrates, grep version 3.1 (https://git.savannah.gnu.org/cgit/grep.git/tree/AUTHORS) was used.

### Antibiotic resistance determination

ABRicate version 1.0.1 (https://github.com/tseemann/abricate)^121^ was used with the CARD^125^, database accessed 4/5/2020.

### Bacteriocin identification

Bacteriocins were identified by downloading the BAGEL4 database^126^ and converting into a multifasta file. The pangenome was then aligned against this using blastp version 2.9.0+^127, 128^ and results were filtered for ≥ 80% coverage and ≥ 50% similarity/positives (to account for different signal sequences).

### Plasmid identification and analysis

Mob-suite version 3.0.0^129^ was used to identify plasmids using the mob recon function, then visualised in ClustVis (https://biit.cs.ut.ee/clustvis/)^124^. No scaling was performed on the data and clustering was performed on rows and columns using the ‘correlation’ distance measure. Putative mobilization of plasmids was determined using the mob_typer function.

### Bacteriophage and prophages identification

The PHASTER (https://phaster.ca/)^130^ web server was used to identify bacteriophage and prophage regions using the API tool. ‘Questionable’ phages (score of 70–90) were grouped with ‘incomplete’.

### Transposon/Insertion sequence identification

ISESCAN version 1.7.2 (https://github.com/xiezhq/ISEScan)^131^ was used to identify transposons and insertion sequences.

### Statistical analysis

*Fisher’s* exact test was performed to identify significance in the proportion of strain types, virulence genes, and plasmids from isolates (unit of analysis) recovered from NQLD, SQLD and VICT. A two sided *p*-value obtained by Monte-Carlo simulation (n = 2000) of at least 0.05 was considered to be significant. Statistical analysis was conducted using stats package implement in R.

## Supplementary Information

Supplementary Information 1.

Supplementary Information 2.

Supplementary Information 3.

Supplementary Information 4.

Supplementary Information 5.

Supplementary Information 6.
