# Supplementary material for: Sequence characterisation and novel insights into bovine mastitis-associated Streptococcus uberis in dairy herds
Source: Sci Rep. 2021 Feb 4;11:3046. doi: 10.1038/s41598-021-82357-3 (PMC7862697; doi:10.1038/s41598-021-82357-3)
Supplement: Supplementary file 1 — Supplementary Information 1. [file 41598_2021_82357_MOESM1_ESM.docx]

**Sequence characterisation and novel insights into bovine mastitis-associated *Streptococcus uberis* in dairy herds**

**Ben Vezina^+^ ^1, 3^, Hulayyil Al-harbi ^2^, Hena R. Ramay ^4^, Martin Soust ^5^, Robert J. Moore ^6^, Timothy W. J. Olchowy ^1,7^ , John I. Alawneh^+^* ^1, 2^**

**^+^** These authors contributed equally.

^1^ Good Clinical Practice Research Group (GCPRG), The University of Queensland, School of Veterinary Science, Gatton, Queensland 4343, Australia

^2^ The University of Queensland, School of Veterinary Science, Gatton, Queensland 4343, Australia

^3^ Centre for Cell Factories and Biopolymers, Griffith Institute for Drug
Discovery, Griffith University, Nathan, Australia

^4^ International Microbiome Centre, Cumming School of Medicine, University of Calgary, Calgary, AB

^5^ Terragen Biotech Pty Ltd., Coolum Beach, Queensland 4573, Australia

^6^ School of Science, RMIT University, Bundoora, Melbourne, 3083 Australia

^7^ Faculty of Veterinary Medicine, University of Calgary, Calgary, Alberta, Canada T3R 1J3

*****John Alawneh, GCPRG, The University of Queensland, School of Veterinary Science, Gatton, Queensland, 4343, Australia, +64 -07-5460 1834, j.alawneh@uq.edu.au


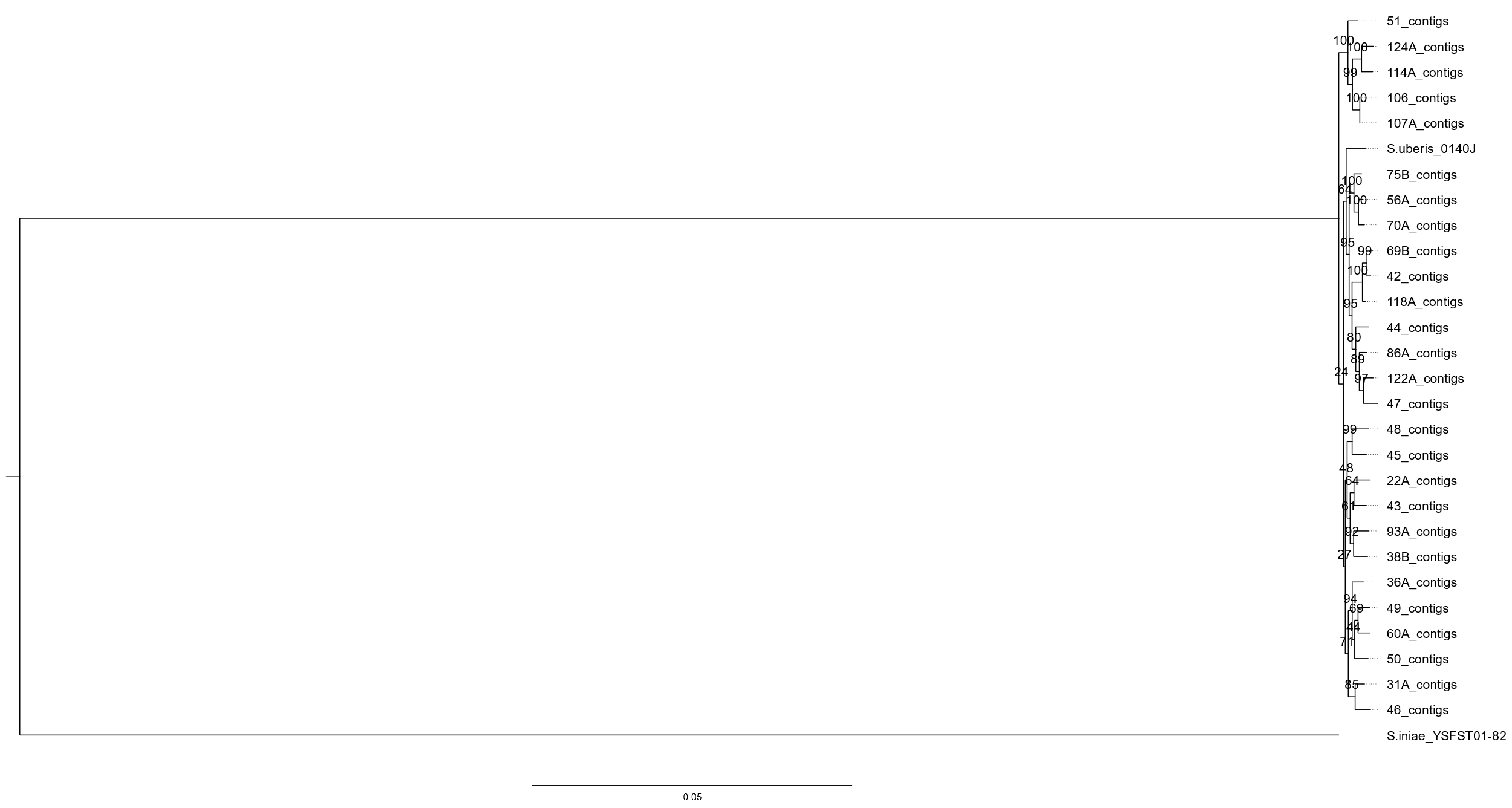


Data S1-1: Maximum likelihood tree phylogenetic tree showing untransformed tree found in Figure 2 (main text). Bootstrap values shown on tree.


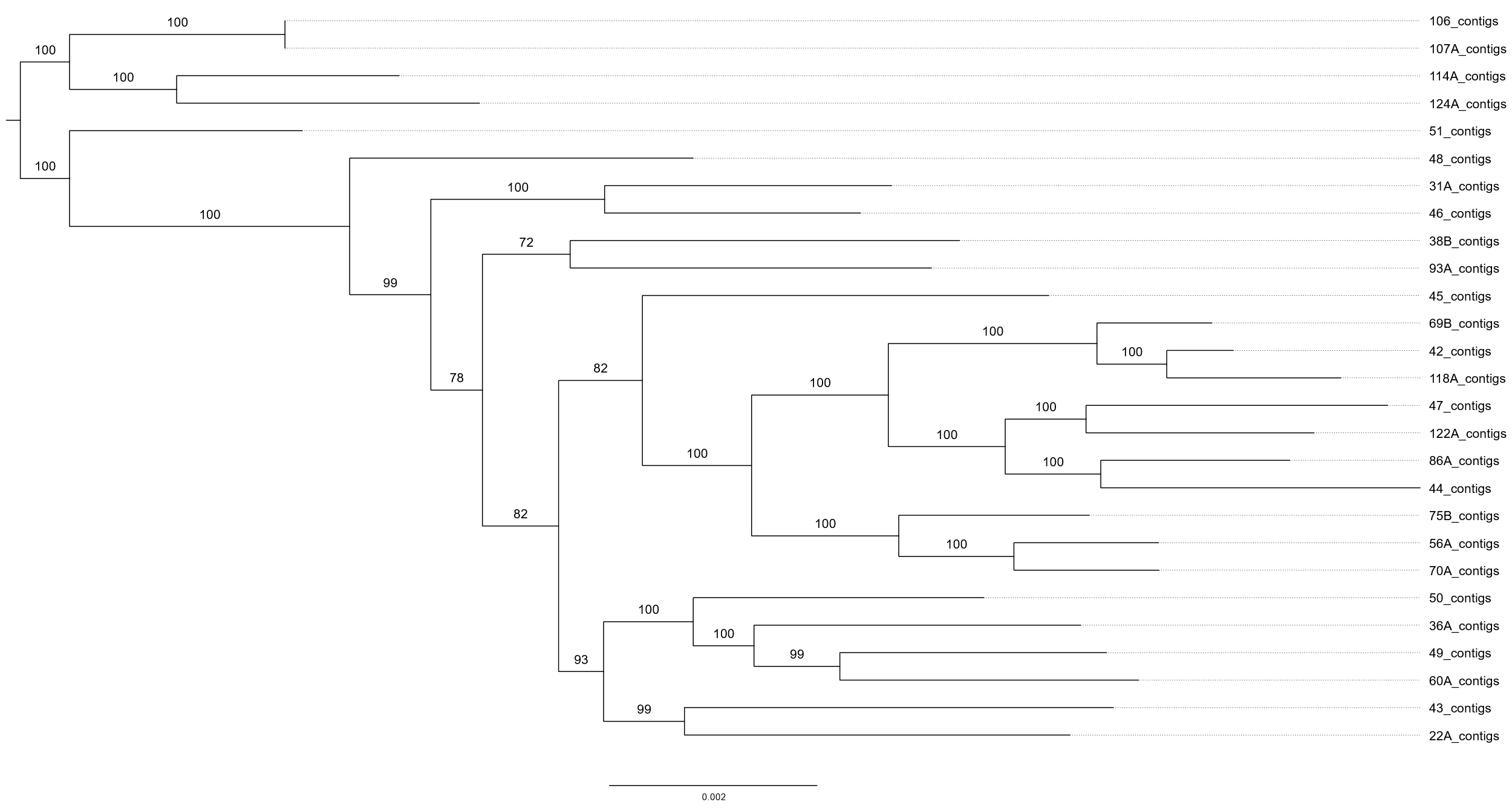


Data S1-2: Maximum likelihood tree phylogenetic tree showing only *S. uberis* isolates identified in this study. Bootstrap values shown on tree.
